# N-Terminal Tails of Histones H2A and H2B Differentially Affect Transcription by RNA Polymerase II In Vitro

**DOI:** 10.3390/cells11162475

**Published:** 2022-08-10

**Authors:** Han-Wen Chang, Alexey V. Feofanov, Alexander V. Lyubitelev, Grigory A. Armeev, Elena Y. Kotova, Fu-Kai Hsieh, Mikhail P. Kirpichnikov, Alexey K. Shaytan, Vasily M. Studitsky

**Affiliations:** 1 Fox Chase Cancer Center, Philadelphia, PA 19111, USA; 2 Biology Faculty, Lomonosov Moscow State University, Moscow 119992, Russia; 3 Department of Molecular Biology, Harvard Medical School, Boston, MA 02114, USA; 4 Shemyakin-Ovchinnikov Institute of Bioorganic Chemistry, Russian Academy of Sciences, Moscow 117997, Russia

**Keywords:** nucleosome, transcription, RNA polymerase II, histone tails, histone H2A, histone H2B, FACT, molecular modeling, spFRET

## Abstract

Histone N-terminal tails and their post-translational modifications affect various biological processes, often in a context-specific manner; the underlying mechanisms are poorly studied. Here, the role of individual N-terminal tails of histones H2A/H2B during transcription through chromatin was analyzed in vitro. spFRET data suggest that the tail of histone H2B (but not of histone H2A) affects nucleosome stability. Accordingly, deletion of the H2B tail (amino acids 1–31, but not 1–26) causes a partial relief of the nucleosomal barrier to transcribing RNA polymerase II (Pol II), likely facilitating uncoiling of DNA from the histone octamer during transcription. Taken together, the data suggest that residues 27–31 of histone H2B stabilize DNA–histone interactions at the DNA region localized ~25 bp in the nucleosome and thus interfere with Pol II progression through the region localized 11–15 bp in the nucleosome. This function of histone H2B requires the presence of the histone H2A N-tail that mediates formation of nucleosome–nucleosome dimers; however, nucleosome dimerization *per se* plays only a minimal role during transcription. Histone chaperone FACT facilitates transcription through all analyzed nucleosome variants, suggesting that H2A/H2B tails minimally interact with FACT during transcription; therefore, an alternative FACT-interacting domain(s) is likely involved in this process.

## 1. Introduction

The eukaryotic genomic DNA is packed to form chromatin that has multiple levels of folding. The first level of folding is a nucleosome that consists of 147 bp DNA tightly wrapped around the histone octamer and the linker DNA connecting octamer-bound DNA regions [1,2,3].

Transcription through chromatin by eukaryotic RNA polymerase II (Pol II) is typically accompanied by the displacement of an H2A/H2B dimer and survival of the remaining DNA-bound histone hexamer (hexasome) in vitro and in vivo [4,5,6,7,8,9]. The nucleosome survival pathway during Pol II transcription (the nucleosomal cycle [5,10]) guarantees the maintenance of chromatin integrity, which is essential for normal cellular functioning [6,7,10]. Transcription through chromatin is also accompanied by Pol II pausing at several positions within the nucleosomes, and this pausing is regulated by multiple protein factors controlling the transcript elongation in vivo [6,7,10]. Similarly, Pol II pauses during transcription through a nucleosome in vitro [11,12].

Histone N-terminal tails and their post-translational modifications are involved in the regulation of Pol II transcription in vitro and in vivo [13,14,15,16]. Removal of H2A/H2B tails, H3/H4 tails, or all tails facilitates pausing relief during Pol II transcription through the nucleosome in vitro [14]. Deletion of the histone H2B Repression (HBR) domain (residues 30–37 within the N-terminal tail of yeast H2B, corresponding to residues 24–31 of *Xenopus laevis* H2B) interferes with Pol II transcription and DNA repair in vivo [17,18,19,20]. Additionally, several proteins, including transcript elongation factors and histone chaperones, strongly affect the efficiency of Pol II traversal through chromatin [11,12,14].

One of the best-studied factors affecting transcript elongation through chromatin is FACT (facilitates chromatin transcription). FACT is a histone chaperone that is involved in multiple cellular processes, including DNA transcription, replication, repair, and cancer development [12,21,22,23,24,25,26,27,28,29,30]. Human FACT is composed of two proteins: SPT16 (suppressor of Ty16) and SSRP1 (structure-specific recognition protein 1) [21]. During Pol II transcription through chromatin in vitro, hFACT strongly affects the rate of transcription and facilitates nucleosome survival, likely transiently interacting with the DNA-binding surfaces of H2A/H2B histone dimers in the nucleosome [12,22]. Structures of several domains of yeast FACT and their complexes with different regions of core histones (see [31] for review) as well as structures of FACT–nucleosome complexes [32,33,34] have been solved. Based on these structural data, the middle and CTD domains of SPT16 and CTD domain of Pob3 (a subunit of yeast FACT that is highly homologous to SSRP1 protein) can interact with the H2A/H2B dimer [35,36]. The middle domain of SPT16 can also bind the H3/H4 tetramer [37]. Human FACT can also bind both the H2A/H2B dimer and the H3/H4 tetramer [38,39]. However, the role of these interactions identified in simple binding assays in vitro has not been determined in any biological process.

Here we used histone mutants to evaluate the role of N-terminal tails of histones H2A and H2B and the interactions of histone H2B with hFACT (identified in a binary binding assay [35] and thought to play a role during FACT-dependent transcription through chromatin [12,35]) in transcription through chromatin. We show that the N-terminal tail of histone H2B plays an important role during Pol II transcription through chromatin by preventing uncoiling of nucleosomal DNA from the histone octamer. Unlike the H2B N-tail, the N-terminal tail of histone H2A likely interacts with the DNA of the neighbor nucleosome and acts as a bridge supporting formation of a dinucleosome containing tailless H2B; however, this results in only partial relief of the nucleosomal barrier to transcription. The putative interactions between the middle domain of SPT16 and histone H2B [35] do not play an important role during FACT-dependent Pol II transcription in vitro.

## 2. Materials and Methods

### 2.1. DNA Templates and Plasmids

All DNA templates were obtained by PCR reaction and gel extraction using a gel extraction kit (Omega Bio-Tek, Norcross, GA, USA) as described in [40,41]. Fluorescent labels (Cy3 and Cy5) were introduced into DNA templates by PCR with the following labeled primers (Lumiprobe, Moscow, Russia):

5′-CCCGGTTCGCGC**[T-Cy3]**CCCGCCTTCCGTGTGTTGTCGTCTCTCGG-3′
5′-ACCCCAGGGACTTGAAGTAATAAGGACGGAGGGCCTCTTTCAACATCGATGCACGG**[T-Cy5]**GGTTAG-3′,

where nucleotides carrying fluorescent labels are bracketed and shown in bold.

The plasmids for expression of recombinant *X. laevis* histones (intact and globular, pET3a plasmid) were obtained from Dr. Karolin Luger [42]. The plasmid for expression of recombinant histones H2BΔ1-31 was constructed by the following procedure. The DNA fragment of H2BΔ1-31 was amplified by a PCR reaction using pET3a-gH2B as a template. The PCR product of H2BΔ1-31 DNA was then ligated to the pET3a plasmid through the ends generated after digestion with restriction enzymes BamH I and NdeI (New England Biolabs, Ipswich, MA, USA) by T4 DNA Ligase (Promega, Madison, WI, USA). All sequences of primers and templates will be provided upon request.

### 2.2. Protein Purification

Yeast RNA polymerase II was purified as described in [43,44]. Recombinant histone H2BΔ1-31, H2A/H2B, H2A/H2BΔ1-31 and gH2A/H2BΔ1-31 histone dimers and histones H3/H4 tetramer were purified and assembled as described in [41,45,46]. Human FACT was purified as described in [12,22]. Trypsinized H2A/H2B dimer (gH2A/gH2B) was purified as described in [14].

### 2.3. Nucleosome Assembly

Nucleosomes were assembled as described in [41]. In short, DNA templates were mixed with purified H2A/H2B dimers and H3/H4 tetramers in the presence of salmon testes DNA as a competitor in the buffer containing 2 M NaCl, 10 mM Tris-HCl (pH 7.4), 0.1% NP-40, and 0.2 mM EDTA. The mixed samples were then dialyzed against buffers with progressively decreasing (2, 1.5, 1, 0.75, 0.5 M, and 10 mM) NaCl at 4 °C for 2 h at each step.

### 2.4. In Vitro Transcription Assay

The in vitro transcription assay with yeast Pol II through a nucleosome was performed as described in [5,12]. In short, DNA/RNA oligonucleotides and purified yeast Pol II were mixed and formed the elongation complexes (ECs). The assembled Pol II ECs were then immobilized on Ni-NTA resins (Qiagen), washed, and eluted from the beads. ECs and nucleosomal templates (or corresponding DNA fragments) were ligated by T4 ligase (Promega). By adding a limited mixture of NTPs and α-^32^P-labeled GTP, Pol II was progressed to position −83 and the RNA was pulse-labeled. Transcription was continued in the presence of unlabeled NTPs, hFACT (final concentration 0.2, 0.4 μM) in the TB buffer (containing 40, 150 or 300 mM KCl) for 10 min. Transcription was terminated using phenol/chloroform extraction. RNA transcripts were purified and analyzed by denaturing PAGE.

### 2.5. Hydroxyl Radical Footprinting

Hydroxyl radicals (OH-) are generated based on the protocol [47]. Ammonium iron (II) sulfate hexahydrate ((NH_4_)_2_Fe(SO_4_)_2_∙6H_2_O) is freshly mixed with EDTA to form 250 µM iron (II) and 500 µM EDTA in the final reaction. In addition, ~100 ng DNA or nucleosomes are incubated with iron (II)-EDTA, sodium ascorbate (to the final 1mM), and hydroxyl peroxide (to the final 1%) in the TB buffer (containing 40 mM KCl but without β-mercaptoethanol) by 50 s at 25 °C. The reaction is stopped by adding glycerol to the final 2%. After digestion, reaction mixtures are separated by native PAGE electrophoresis, and DNA is isolated from the gel and analyzed by the denaturing PAGE.

### 2.6. Single-Particle Förster Resonance Energy Transfer Microscopy

Core nucleosomes were assembled using 603 DNA template fluorescently labeled at positions +13 and +91 bp with Cy3 and Cy5 fluorophores, respectively, and then purified by non-denaturing PAGE. The single-particle Förster resonance energy transfer (spFRET) measurements were performed in the buffer (10 mM Tris-HCl pH 8.0, 0.5 mM EDTA) containing 0.15 M KCl as described in [27]. For the time course analysis at higher ionic strength, nucleosomes were diluted to a concentration of 1 nM in the buffer with 0.5 M KCl and measured by spFRET microscopy during the time periods of 0–7.5, 7.5–15, and 15–22.5 min after dilution [27,48].

The spFRET microscopy measurements and proximity ratio (E_PR_) calculations were performed as described earlier [49]. The relative frequency distributions of nucleosomes by E_PR_ were calculated using data collected from >1000 individual nucleosomes in each independent measurement. Each E_PR_-profile was fitted with a sum of two Gaussians describing particular conformational states of nucleosomes. The fractions of nucleosomes in the different states were calculated as the areas under corresponding Gaussian peaks normalized to a total area of a plot. Mean values and standard errors of mean were calculated from three or more independent experiments.

### 2.7. Single-Particle Fluorescence Intensity Analysis

To study nucleosome dimerization, single-particle analysis of fluorescence intensities of nucleosomes was performed in solution and in gel. In solution, fluorescently labeled nucleosomes were measured as described above, but direct excitation of Cy5 was conducted with the 633 nm wavelength, and fluorescence was recorded in the 650–800 nm range.

For the analysis in gel, a gel obtained after electrophoresis was fixed between object and cover glasses and subjected to single-particle fluorescence intensity measurements under a microscope in the following way. A fluorescent image of the gel obtained with Amersham Typhoon RGB imager (Cytiva, Emeryville, CA, USA) was used to find positions of bands containing nucleosomes. An area of the band, where concentration of nucleosomes was low enough for a single particle measurement, was subjected to the analysis with the excitation at 633 nm and detection in the 650–800 nm range. Recording of single-particle signal intensities was conducted with a change of laser focus position along the band by ~10 μm every 10 s.

Time records of single photons were analyzed with FretBursts software [50] to assess the background level and detect fluorescence bursts. The burst searching window was set to 10 successive photons. The bursts were accepted if they had more than 30 photons, and their peak brightness was at least three times higher than the average background level. Finally, each single nucleosome particle measured in solution or in gel was characterized by Cy5 fluorescence intensity (I_Cy5_), and a relative frequency distribution of I_Cy5_ values was calculated for each experiment involving 2000–3000 particles.

## 3. Results

### 3.1. H2A N-Tail Mediates Internucleosomal Interactions

**Figure 1:**
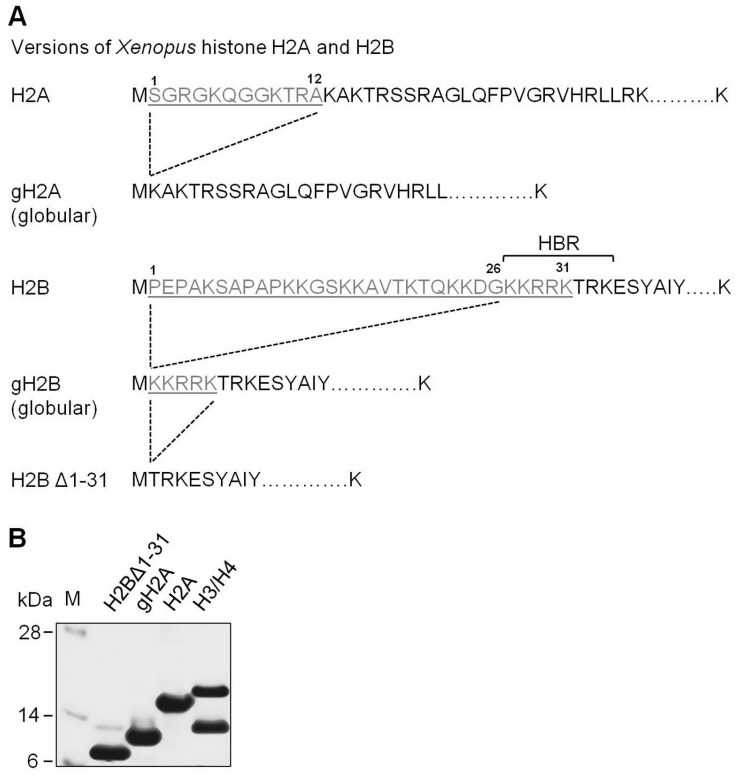
Experimental system. (**A**) Partial amino acid sequences of intact and mutant versions of histones H2A and H2B used in this work. HBR region of histone H2B is indicated. (**B**) Analysis of purified proteins by SDS-PAGE (staining with Coomassie). M: molecular mass markers.

To evaluate the role of N-terminal tails of histones H2A and H2B in nucleosome structure and transcription through chromatin, nucleosomes were assembled on the well-characterized 603 nucleosome positioning DNA sequence [4,40] using full-length H3/H4 tetramers and one of the following four types of H2A/H2B dimers: full-length H2A/H2B, globular H2A/H2B (gH2A/gH2B containing deletion of amino acids 1–26 in histone H2B), H2A/H2BΔ1-31, or gH2A/H2BΔ1-31 (Figure 1).

**Figure 2:**
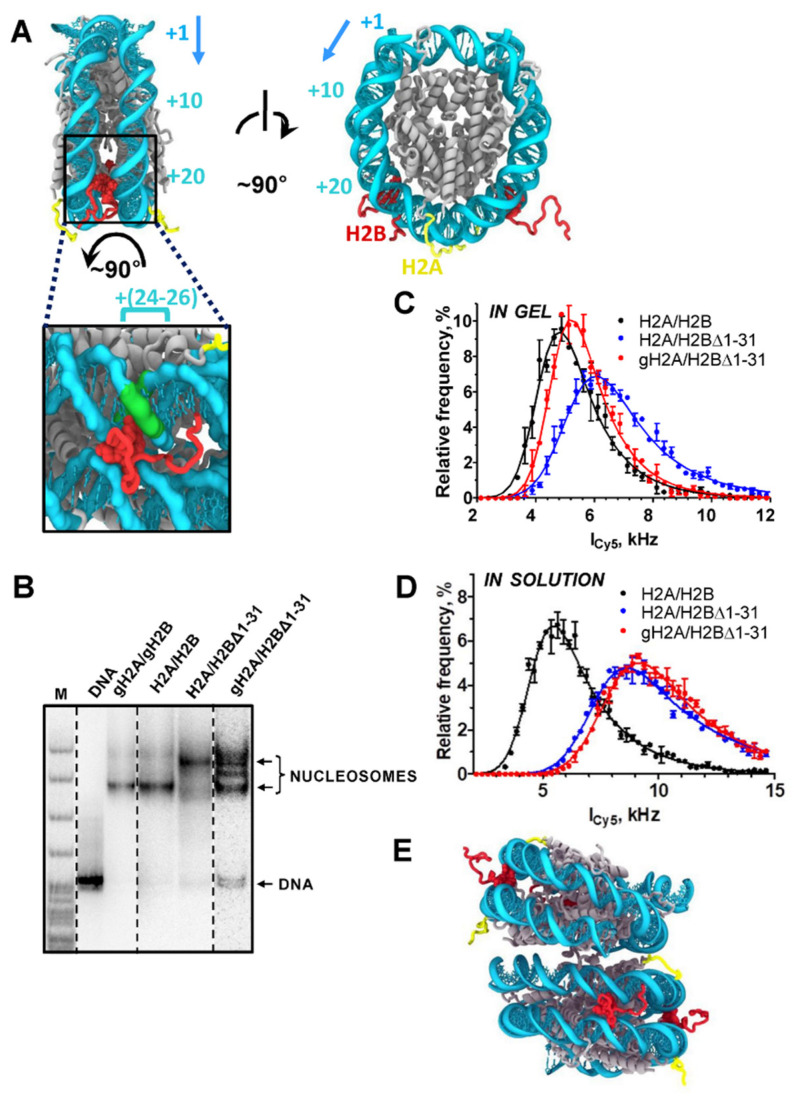
H2A tails mediate the interaction between the DNA gyres of neighboring nucleosomes. (**A**) Locations of H2B and H2A N-terminal tails in the nucleosome core particle (PDB 1KX5, lateral and side views). DNA is colored in cyan, histones—in grey, H2B N-terminal tails (positions 1–31)—in red, H2A N-terminal tails (position 1–12)—in yellow. H3 N-terminal tails are truncated for clarity. All atoms of the important KKRRK motif of the H2B tail (positions 27–31) are shown as van der Waals spheres. The H2B tail mediates the interaction between two DNA gyres within the same NCP. Histone H2B tails interact with nucleosomal DNA at the distance of ~25 bp (+25) from the nucleosomal boundary. Direction of transcription is indicated by an arrow. A zoom up view is provided with the DNA region +(24–26) highlighted in green. (**B**) Analysis of gel-purified nucleosomes by native PAGE. Nucleosomes contained intact H3/H4 histones and various variants of H2A/H2B histones. M: pBR322-MspI digest. (**C**,**D**) Distributions of single nucleosomal particles by fluorescence intensity (I_Cy5_) in gel (**C**) and in solution (**D**). (**E**) Location of H2A N-terminal tails in the crystal packing of nucleosome core particles (NCP, PDB 1KX5). Two NCPs stacked by their lateral surfaces are shown, with H2A N-tails mediating the interaction between the DNA gyres of neighboring NCPs. The color code is in panel (**A**).

In the nucleosome structure, the H2A N-tails are localized on the lateral surfaces of the nucleosome core particle (NCP), which is very different from the H2B N-tails positioned between the two DNA gyres (Figure 2A). Thus, the functional roles of the H2A and H2B N-tails are likely different from each other. To evaluate the roles of the tails, different mutant nucleosomes were analyzed by native gel electrophoresis (Figure 2B). The gH2A/gH2B and H2A/H2B nucleosomes have similar mobilities in the gel. However, the H2A/H2BΔ1-31 nucleosome has a different, lower mobility in the gel that could be explained by either (a) nucleosome unfolding [27] or (b) nucleosome dimerization. Since nucleosome unfolding results in a dramatic change in the pattern of hydroxyl radical footprinting [51], this method could be used to discriminate between two possibilities. The footprinting profiles of H2A/H2BΔ1-31 and H2A/H2B-containing nucleosomes are nearly identical (Supplementary Figure S1), suggesting that H2A/H2BΔ1-31 nucleosomes are not unfolded and tend to dimerize.

To further evaluate this possibility, nucleosomes separated by electrophoresis were studied by single particle fluorescence microscopy in gel. The fluorescence of Cy3-Cy5-labeled nucleosomes was measured after direct Cy5 excitation, and distributions of single nucleosomal particles by fluorescence intensity were compared (Figure 2C). For different monomeric nucleosomes, these distributions are expected to be similar, whereas nucleosome dimers should have a distribution shifted towards higher fluorescence intensity due to the presence of two Cy5 labels per particle. Fluorescence intensities of single H2A/H2BΔ1-31 particles were noticeably higher than those of single H2A/H2B nucleosomes, in agreement with the proposal of dimerization of H2A/H2BΔ1-31 nucleosomes. Fluorescence intensities of single gH2A/H2BΔ1-31 particles were intermediate between H2A/H2B and H2A/H2BΔ1-31 particles, indicating the propensity of gH2A/H2BΔ1-31 to dimerize (Figure 2C). Similar measurements in solution revealed higher fluorescence intensities of single H2A/H2BΔ1-31 and gH2A/H2BΔ1-31 particles as compared to H2A/H2B nucleosomes (Figure 2D), indicating that gH2A/H2BΔ1-31 nucleosomes are more efficiently dimerized in solution than in gel. The electrophoresis data (Figure 2B) and the data obtained by single-particle fluorescence microscopy (Figure 2C,D) are consistent and indicate the following order in the efficiency of dimerization of nucleosomes containing H2A/H2BΔ1-31 > gH2A/H2BΔ1-31 > H2A/H2B.

The nucleosome dimerization is partially reversed in gel by deletion of the H2A N-tail (gH2A/H2BΔ1-31 nucleosome, Figure 2B), suggesting that the H2A N-tail likely participates in the interaction between the nucleosomes. Indeed, analysis of crystal packing of nucleosome core particles shows that H2A N-tails mediate the interaction between the DNA gyres of neighboring NCPs (Figure 2E).

### 3.2. Histone H2B N-Tail Stabilizes the Nucleosome Structure

**Figure 3:**
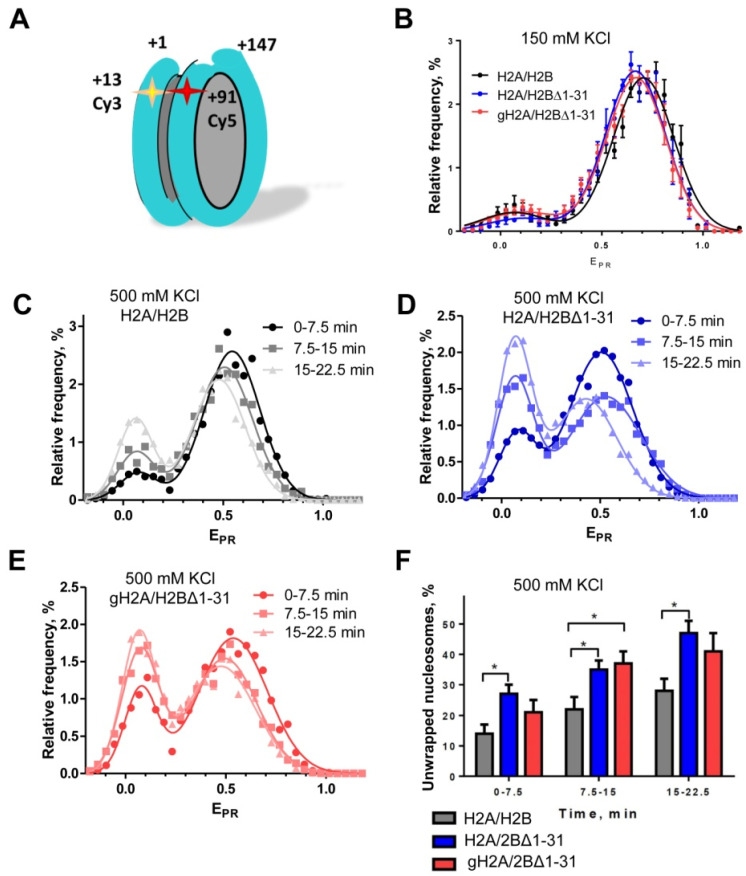
Histone H2B N-tail stabilizes the nucleosome structure. Nucleosomes containing H2A/H2B, H2A/H2BΔ1-31 or gH2A/H2BΔ1-31 histones were studied by spFRET microscopy at 150 mM KCl (**A**) or 500 mM KCl (**B**–**E**). (**A**) A schematic diagram of the nucleosome labeled with Cy3 and Cy5 dyes (asterisks). (**B**) Frequency distributions of nucleosomes by E_PR_ at 150 mM KCl. (**C**–**E**) Typical frequency distributions of nucleosomes by E_PR_ at 500 mM KCl as a function of time: NCP with H2A/H2B (**C**), NCP with H2A/H2BΔ1-31 (**D**) and NCP with gH2A/H2BΔ1-31 (**E**). (**F**) Relative content of nucleosomes with uncoiled DNA (low-E_PR_ subpopulation) at 500 mM KCl as a function of time (mean ± SEM, *n* = 3–5, * *p* < 0.05). Note that both H2A/H2BΔ1-31 and gH2A/H2BΔ1-31 nucleosomes contain more uncoiled DNA than the H2A/H2B nucleosomes.

Core histone tails carry a significant net positive charge that neutralizes the negative charge of nucleosomal DNA, thus reducing the Coulomb repulsion between adjacent DNA gyres in the nucleosome and possibly stabilizing the nucleosome core particle (NCP). Deletion of core histone tails could reduce the affinity of nucleosomal DNA to core histones, making nucleosomal DNA more accessible. To evaluate the effect of histone tail deletion on NCP structure and stability, spFRET measurements of fluorescently labeled nucleosomes (Figure 3A) containing H2A/H2B, H2A/H2BΔ1-31, or gH2A/H2BΔ1-31 histones were performed at 150 and 500 mM KCl. Then, the proximity ratios (E_PR_) were calculated to determine changes in the distance between the labeled DNA sites through changes in FRET efficiency [27]. At the physiological concentration of KCl (150 mM), the frequency distributions of the analyzed nucleosomes (E_PR_) are characterized by the presence of two separate maxima at E_PR_ = 0.05 and E_PR_ = 0.67 ÷ 0.70 (Figure 3B). The vast majority of NCPs demonstrated a higher E_PR_ value—an indicator of tightly wrapped nucleosomal DNA. The E_PR_-profiles of NCP with non-modified and modified histones are very similar, indicating that dimerization of H2A/H2BΔ1-31 or gH2A/H2BΔ1-31 does not affect packing of nucleosomal DNA, at least near the edge of NCPs where Cy3 and Cy5 labels are placed. Since packing of DNA is similar in nucleosomes containing different versions of histones H2A and H2B, it is highly unlikely that any of these nucleosomes selectively miss H2A/H2B dimers or form alternative nucleosome structures such as hemisomes [52,53].

To evaluate the effect of H2BΔ1-31 and gH2A/H2BΔ1-31 deletions on nucleosome stability, NCPs were subjected to ionic strength-induced unfolding at 500 mM KCl. Changes in NCP structure were studied using spFRET microscopy during three sequential, identical time periods at 500 mM KCl. Comparison of E_PR_ profiles calculated for these time periods revealed an increase in the low-E_PR_ peak and a slight shift of the high-E_PR_ peak maxima to lower values, indicating time-dependent uncoiling of nucleosomal DNA (Figure 3C–E). This uncoiling is facilitated by a high ionic strength and occurs most probably during collisions of nucleosomes with a glass surface of the measuring cell, leading to dissociation of some histones (for example, H2A/H2B dimer(s)) from nucleosomes. In this case the rates of DNA uncoiling depended on the stability of nucleosomes and were different for NCPs containing H2A/H2B, H2A/H2BΔ1-31 or gH2A/H2BΔ1-31 histones (Figure 3C–E).

To quantify and quantitatively compare the rates of uncoiling of nucleosomal DNA, the fractions of low-E_PR_ particles were calculated for each measured time period (Figure 3F). Incorporation of H2BΔ1-31 and gH2A/H2BΔ1-31 histones in nucleosomes increased the rates of DNA uncoiling as compared with H2A/H2B-containing nucleosomes (Figure 3C–F), suggesting that the histone tails protect nucleosomes from disruption at 500 mM KCl. Deletion of H2A tails does not further destabilize nucleosomes containing H2BΔ1-31, suggesting that H2A tails play a less important role in preventing DNA uncoiling than H2B N-tails.

In summary, the E_PR_-profiles indicate that replacement of H2A/H2B dimers with H2A/H2BΔ1-31 or gH2A/H2BΔ1-31 results in only minor changes in the structure of the nucleosomes but considerably affects nucleosome stability. In particular, deletion of the 1–31 region of histone H2B (but not H2A N-tail) strongly facilitates uncoiling of nucleosomal DNA at 500 mM KCl, suggesting that this deletion could facilitate uncoiling of nucleosomal DNA from the octamer during transcription.

### 3.3. Nucleosomal Pausing at the +(11–15) Region Is Partially Relieved by Deletion of H2B N-Tail

**Figure 4:**
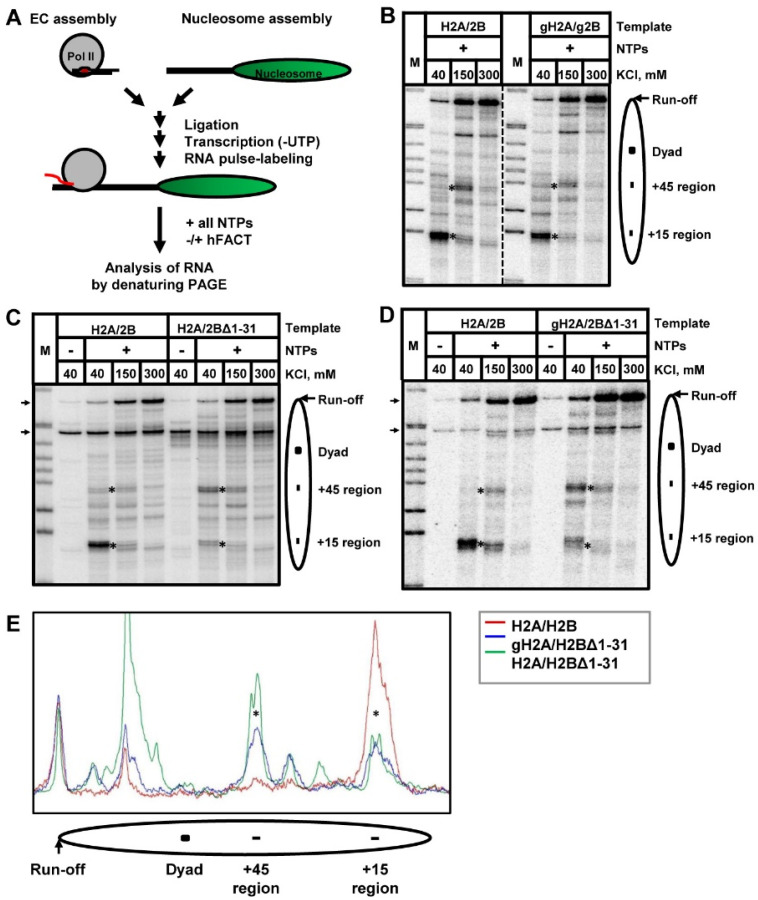
Deletion of H2B N-tail results in partial relief of the +15 nucleosomal pausing during Pol II traversal through a nucleosome. (**A**) The experimental approach. An elongation complex (EC) was assembled using purified yeast Pol II and a set of DNA and RNA oligonucleotides [5,12]. The assembled EC was ligated to 603 DNA or a nucleosome to obtain EC-119 (EC stalled 119 bp upstream of the nucleosomal boundary). After ligation, while Pol II was “walked” and stalled at the position -83, the RNA was pulse-labeled in the presence of [α-^32^P] GTP and the subset of NTPs. Then, transcription was resumed by addition of all unlabeled NTPs in the presence or absence of FACT. In addition, 603 nucleosomes containing full length H2A/H2B, or gH2A/gH2B (**B**), or H2A/H2BΔ1-31 (**C**), or gH2A/H2BΔ1-31 (**D**), dimers were transcribed by Pol II at the indicated concentrations of KCl. Analysis of pulse-labeled RNA was performed by denaturing PAGE. As expected [14], the replacement of H2A/H2B with gH2A/gH2B only minimally affects the nucleosomal pausing. In contrast, the presence of solely H2BΔ1-31 results in a strong relief of the nucleosomal pausing at the +15 bp region during Pol II transcription. In the presence of gH2A/H2BΔ1-31, the nucleosomal pausing at the +15 bp region is partially relieved during Pol II transcription. M: *Msp*I-digested pBR322 (NEB). Asterisks indicate the positions of the +15 and +45 regions of pausing. Arrows indicate the positions of labeled DNA fragments (ligated and not ligated) present in some nucleosome preparations. (**E**) Quantitative analysis of nucleosomal pausing after Pol II transcription. The pausing patterns obtained after Pol II transcription through H2A/H2B-, gH2A/H2BΔ1-31-, and H2A/H2BΔ1-31-containing nucleosomes (red, blue, and green lines, respectively) at 40 mM KCl were scanned along the lines using OptiQuant software, aligned, and normalized by the yield of run-off transcripts.

To evaluate the effect of N-terminal “tails” of histones H2A and H2B on transcription through chromatin, nucleosomes containing H2A/H2B, globular H2A/H2B, H2A/H2BΔ1-31, or gH2A/H2BΔ1-31 were transcribed in vitro using a “minimal” experimental system (Figure 4A) that recapitulates many features of chromatin transcribed in vivo [4,5,9,11,12,54,55]. In short, authentic elongation complexes were assembled using purified yeast Pol II and a set of DNA and RNA oligonucleotides [11,43,44]. The assembled elongation complexes were ligated to the 603 DNA or nucleosomes. The elongation complexes were extended in the presence of a partial combination of NTPs and P^32^-labeled γ-GTP to form stalled EC-83 (in this elongation complex the active center of Pol II is positioned 83 bp upstream of the nucleosomal boundary). Transcription was continued by adding an excess of all unlabeled NTPs in the presence or absence of human FACT.

As expected, Pol II pauses primarily at the positions +(11–15) and +(45–48) bp (relative to the proximal boundary of the nucleosome) during transcription through a single, well-positioned nucleosome (Figure 4B). The earlier pauses are more pronounced when transcription is performed in the presence of a lower concentration of KCl, whereas all pauses are more efficiently relieved in the presence of a higher concentration of KCl (e.g., at 300 mM KCl). As expected, the pausing patterns formed during transcription through the nucleosome containing gH2A/gH2B dimer and through the intact nucleosome are similar (Figure 4B) [14]. Intriguingly, incorporation of H2BΔ1-31 mutant results in strong relief of the nucleosomal pausing at the +(11–15) region during Pol II transcription; the relief is most apparent at 40 and 150 mM KCl (Figure 4C). The relief of the +(11–15) pausing is accompanied by an increase of pausing at the +45 region. The data indicate that deletion of amino acids 1–31 of histone H2B selectively reduces only the +(11–15) nucleosomal barrier, likely because it weakens the interactions between DNA and the histone octamer close to the boundary of the nucleosomal DNA.

Since the deletion of amino acids 1–26 of histone H2B N-tail has only a minor effect on transcription in the presence of gH2A (Figure 4B), the data suggest one of the following non-exclusive scenarios: (1) the presence of 27–31 region of histone H2B is critical for the observed effect on transcription through the nucleosome and/or (2) the presence of intact histone H2A is required for the inhibitory effect of the histone H2B N-tail. In order to evaluate these two hypotheses, we assembled the nucleosome with a gH2A/H2BΔ1-31 mutant dimer and compared the pausing pattern of Pol II transcription to the nucleosome containing wild-type histones (Figure 4D). The results for gH2A/H2BΔ1-31 and H2A/H2BΔ1-31 nucleosomes are similar—in both cases the +(11–15) pausing is relieved together with the increase of +45 pausing (Figure 4), although in the first case the relief of pausing is less pronounced (Figure 4E). Thus, the data suggest that the presence of the 27–31 region of histone H2B dictates the efficiency of Pol II transcription through the +(11–15) region of the nucleosome.

In addition to relief of the +(11–15) nucleosomal barrier, the presence of H2BΔ1-31 in nucleosomes results in other changes in the pausing pattern, both before and after the nucleosomal dyad. The majority of these changes are likely explained by the relief of the +15 pausing and corresponding changes in the intensity of the pausing further in the nucleosome. However, in some cases the presence of H2BΔ1-31 results in the appearance of strong Pol II pausing behind the dyad (Figure 4E) that is difficult to explain solely by the decrease in the intensity of +(11–15) pausing. Further experiments are required to evaluate the nature of the pausing behind the dyad observed in case of nucleosomes containing H2A/H2BΔ1-31 (Figure 4E).

The 27–31 region of H2B is localized between and likely interacts with the two DNA gyres (Figure 2A). These interactions stabilize the structure of a nucleosome, particularly the region of nucleosomal DNA localized ~25 bp from the nucleosomal boundary (Figure 2A) [56] that forms strong DNA–histone interactions [57]. In agreement with this proposal, the nucleosome lacking the 27–31 region of histone H2B is less stable and the nucleosomal DNA can more easily be uncoiled at 500 mM KCl (Figure 3). Since transcribing Pol II covers ~35 bp DNA around the active center of the enzyme, the +25 DNA region needs to be uncoiled from the octamer when Pol II proceeds through the +(11–15) region [10]. Thus, our data suggest that deletion of the 27–31 residues of histone H2B likely results in disruption of the DNA–histone interaction at the +25 region and facilitates Pol II progression through the +(11–15) region.

### 3.4. Deletion of H2B N-Tail Does Not Inhibit hFACT Action during Pol II Transcription through a Nucleosome

**Figure 5:**
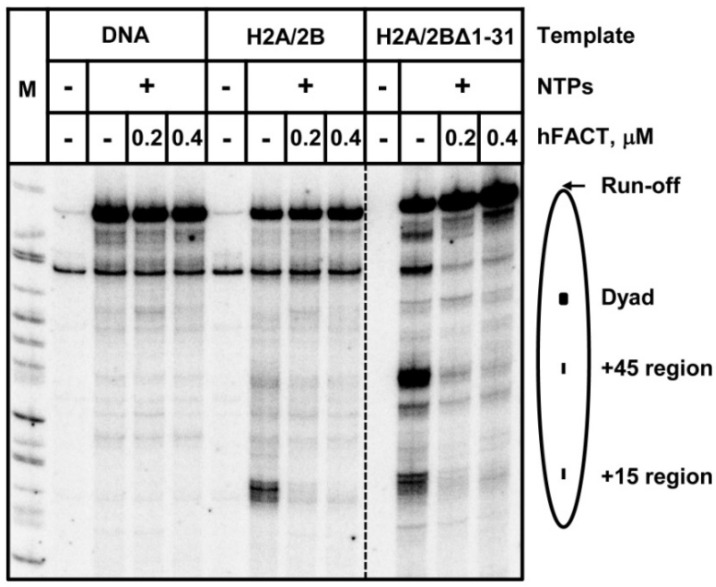
Deletion of H2B N-tail is not sufficient to inhibit FACT-facilitated transcription in vitro. In fact, 603 DNA or nucleosomes, which contained H2A/H2B or H2A/H2BΔ1-31 dimers, were transcribed by Pol II at 150 mM KCl in the presence or absence of human FACT protein complex. FACT strongly facilitates Pol II transcription through a nucleosome in vitro. This activity of FACT is not affected by the deletion of the H2B tail.

Our previous work suggested that FACT affects transcription through a nucleosome by transiently interacting with the DNA-binding surface of an H2A/H2B dimer that becomes transiently exposed during DNA uncoiling from the histone octamer that occurs during transcription [12]. Since FACT can interact with the N-terminal tail of histone H2B [35,36], the mutant H2BΔ1-31 was expected to inhibit hFACT action during Pol II transcription through chromatin. To evaluate this proposal, we performed the in vitro Pol II transcription using intact or H2BΔ1-31 mutant 603 nucleosomes in the presence or absence of hFACT (Figure 5). As expected, pausing at all regions on the intact nucleosomal DNA is relieved in the presence of hFACT (Figure 5). H2BΔ1-31 nucleosomes are characterized by a pausing pattern that is considerably different from other analyzed nucleosomes (Figure 4 and Figure 5); nevertheless, FACT efficiently facilitates transcription through all pausing regions. Furthermore, we also analyzed another histone H2B mutant, H2BI36E, which has a lower affinity to SPT16 in comparison with intact H2B [35]. It also does not affect FACT-dependent transcription (Supplementary Figure S2).

Thus, both deletion of the H2B N-tail and the H2BI36E mutation, which strongly affect the putative FACT–H2B interaction, have no effects on hFACT action during Pol II transcription through the nucleosome, suggesting that an alternative FACT-interacting domain(s) of the H2A/H2B dimer is involved in this process.

### 3.5. A Model: The Roles of H2B and H2A N-Tails during Pol II Transcription

**Figure 6:**
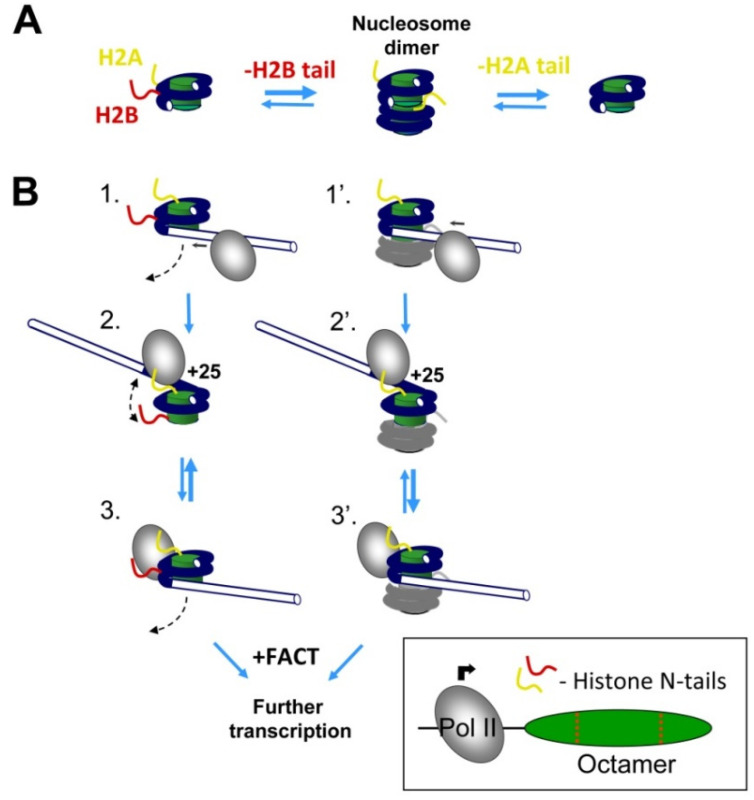
A model: the role of N-terminal tails of histones H2A and H2B during Pol II transcription. (**A**) N-terminal tails of histones H2A and H2B strongly affect nucleosome dimerization. Only one histone tail per nucleosome is shown for clarity. (**B**) Effect of histone tails on transcription by Pol II. When Pol II approaches a nucleosome (intermediates 1 and 1′), the nucleosomal DNA in front of Pol II is partially and transiently uncoiled from the histone octamer (intermediates 2 and 2′); only the uncoiled state is permissive for Pol II progression along the +(11–27) region of nucleosomal DNA [5,58,59]. The promoter-proximal N-tail of histone H2B stabilizes the contacts of nucleosomal DNA to the histone octamer at the +25 region (intermediates 3 and 3′), shifting the equilibrium between the coiled and uncoiled states, and inhibiting Pol II passage through this region. In the presence of FACT, the equilibrium is shifted towards the uncoiled state [12], favoring more efficient transcription. Although removal of the N-tail of histone H2B facilitates formation of nucleosome dimers, this dimerization only minimally affects transcription through the nucleosome.

Although removal of the N-tail of histone H2B facilitates formation of nucleosome-nucleosome dimers, and additional removal of the H2A tail prevents dimer formation (Figure 6A), the nucleosome–nucleosome interactions *per se* only minimally affect the efficiency of transcription through the nucleosome. The data suggest that removal of the H2B tail strongly facilitates transcription through the +(11–15) region of nucleosomal DNA through a different mechanism involving facilitated uncoiling of nucleosomal DNA (Figure 6B).

Previously it has been shown that during transcription through the +(11–15) region, DNA behind Pol II is transiently uncoiled from the histone octamer, although it can form several transient intranucleosomal DNA loops later during transcription that eventually result in nucleosome recovery in vitro [5]. It is likely that only the uncoiled state is permissive for Pol II progression along the +(11–27) region of nucleosomal DNA [5]. When Pol II transcribes through the +(11–15) region of a nucleosome containing intact H2B, it pauses and continues the progression, capturing the uncoiled DNA state.

Accordingly, we propose that the +(11–15) nucleosomal pausing highly depends on the equilibrium between the coiled/uncoiled states of nucleosomal DNA (Figure 6B). The positively charged N-terminal tail of histone H2B interacts with DNA and likely shifts the equilibrium towards a more coiled state. Indeed, deletion of the positively charged region in the H2BΔ1-31 mutant likely reduces the overall positive charge and weakens the DNA–histone interactions. As a result, the region of nucleosomal DNA bound to the H2A/H2B dimer is more easily uncoiled from the H2BΔ1-31 histone octamer than from the intact octamer (Figure 3C–F), and transcription through the +(11–15) region by Pol II occurs more efficiently (Figure 4). Furthermore, the +(11–15) pausing relief by Pol II is much more pronounced in the H2BΔ1-31 mutant than in the gH2B mutant, suggesting that the residues 27–31 of H2B are particularly important. The structural analysis reveals that region 27–31 of H2B stabilizes two DNA gyres and interacts with 24–26 bp of nucleosomal 603 DNA (Figure 2A and Figure 6B), which localizes at one of the high-affinity DNA-histone interacting regions of the 603 nucleosome [10,57]. Thus, the DNA–histone interactions within the H2B region are likely critical for the +(11–15) pausing during Pol II transcription.

## 4. Discussion

Taken together, our data suggest that one of the H2A N-tails can interact with nucleosomal DNA of another nucleosome and thus facilitate nucleosome–nucleosome interactions (Figure 2B,C). However, these internucleosomal interactions only minimally affect the efficiency of transcription through the nucleosome (Figure 4 and Figure 5). Similarly, deletion of the 1–26 region of histone H2B does not considerably affect transcription through the nucleosome (Figure 4B). In contrast, further deletion of the N-terminal tail of H2B (region 1–31), including the 27–31 region that interacts with the +(24–26) region of nucleosomal DNA (Figure 2A), results in uncoiling of nucleosomal DNA at 500 mM KCl (Figure 3) and a strong reduction of the +(11–15) nucleosomal barrier during Pol II transcription through chromatin (Figure 4). Deletion of the N-terminal tail of histone H2A partially reverses this pausing relief (Figure 4D) but has only a minimal effect on the uncoiling of nucleosomal DNA (Figure 3). None of the H2A/H2B tails has any effect on FACT-dependent transcription (Figure 5). Accordingly, the data suggest that the amino acids 1–31 of the H2B N-tail, most likely the amino acids 27–31, are critically involved in the +(11–15) nucleosomal pausing during the Pol II transcription through chromatin (Figure 6). Deletion of the 1–31 region of H2B results in relief of +(11–15) pausing during the Pol II transcription, likely because deletion of the positively charged region in the 27–31 region results in less strong interactions between DNA and histones, thus facilitating transition from the coiled to uncoiled state of nucleosomal DNA and facilitating transcription (Figure 6).

The early studies have demonstrated that +(11–15) pausing in Pol II transcription is sequence dependent, suggesting that it could depend on the strength of DNA–histone interactions [5,10,11]. There are three regions of strong DNA–histone interactions within the 603-nucleosome positioning sequence. One of them is localized at the position +(13–27) [10,57]. Previous structural studies suggested that deletion of the 1–26 region of H2B weakens the interactions between the +(13–27) region of nucleosomal DNA with the residues 30–33 of H2B [56]. However, deletion of the 1–26 region of H2B does not affect the +(11–15) pausing during Pol II transcription (Figure 4D), suggesting that the structure in solution and the crystal structure could be slightly different.

Our experiments indicate that during transcription of H2A/H2BΔ1-31- and gH2A/H2BΔ1-31-containing nucleosomes, a lower +(11–15) barrier was detected (Figure 2A and Figure 4D). Thus, the presence of the 27–31 region of histone H2B is critical for the occurrence of +(11–15) pausing during Pol II transcription through a nucleosome containing intact histone H2B. In the nucleosome, residues 27–31 of H2B interact with the +(24–26) high-affinity DNA region (Figure 2A). These interactions are absent in the nucleosome containing H2BΔ1-31 mutant. The efficiency of DNA uncoiling in the H2BΔ1-31 nucleosome at the 0.5 M KCl buffer is also increased in comparison with the intact nucleosome (Figure 3), suggesting that deletion of the region 1–31 of H2B likely facilitates uncoiling of nucleosomal DNA from the histone octamer during transcription. Thus, our results suggest that the interaction between the H2B N-tail and the +(13–27) DNA region is critical for the stability of the nucleosome and the +(11–15) pausing during Pol II transcription through the nucleosome (Figure 6). Previously, we have observed that the +(11–15) pausing is also decreased in the gH3/gH4-containing nucleosomes [14], likely because the H3 N-tail interacts with the DNA at the entry/exit region of nucleosomal DNA [56]. Taken together, our data suggest that uncoiling of the +(11–27) region of nucleosomal DNA during transcription is affected by DNA sequence and by interactions of the N-tails of histones H3 and H2B with the +(11–27) DNA region (Figure 6).

Histone N-tails form inter- and intranucleosomal interactions with DNA through their positively charged amino acids [15,60,61]. These DNA–histone interactions stabilize the structure of the nucleosome and the folding of chromatin. Here, we demonstrate that the KKRRK motif of the H2B tail (region 27–31) interacts with nucleosomal DNA and thus stabilizes the nucleosome (Figure 2A and Figure 3). This KKRRK motif also significantly affects the efficiency of Pol II transcription through the nucleosome (Figure 6). Since the deleted KKRRK motif is a part of the HBR domain (Figure 1A) and this deletion causes changes in transcription through the nucleosome and nucleosome destabilization, our data are consistent with the results of the previous study showing that deletion of the HBR domain of histone H2B causes abnormal transcription and genome instability in the yeast [17,18,62]. This partial disruption of the HBR domain was also expected to affect the interaction of H2B with FACT and therefore affect FACT-dependent transcription [62]; however, this effect was not observed in our system in vitro (Figure 5).

Previous studies have demonstrated that during chromatin folding, the N-terminal tail of histone H4 likely interacts with the acidic patch on H2A [60]. Here, we identified another possible internucleosomal interaction between DNA and the H2A N-tail. We have determined that the H2A N-tail stabilizes internucleosomal interactions, likely interacting with the DNA on the surface of the neighbor nucleosome (Figure 2B,C) and contributing to the chromatin folding in the nuclei. The H2A-dependent nucleosome dimerization is pronounced when the 1–31 region of H2B is removed (Figure 2B), suggesting that the higher exposure of nucleosomal DNA in the H2B mutant nucleosome promotes the dimerization (Figure 6A).

Why doesn’t the deletion of the N-terminal tail of H2B strongly affect FACT-dependent transcription? The interaction between the middle domain of SPT16 and histone H2B, as well as interaction between the HBR domain and FACT, were identified in binary-binding assays [17,18,35,62] and were thought to play a role during FACT-dependent transcription through chromatin [12,62]. However, since FACT can interact with different regions in the histone octamer through different domains [35,36,37,38,39,63], the lack of FACT–H2B interaction could be compensated by another FACT–octamer interaction during FACT-dependent transcription. Furthermore, the interaction between the HBR domain and FACT was not observed in the recently solved structures of FACT–nucleosome complexes where the C-terminal tail of Spt16 subunit interacts with the H2A/H2B dimer through a different surface [32,33,34]. It remains to be established whether the HBR domain plays any role during FACT-dependent transcription in vivo.

## Data Availability

The data presented in this study are available on request from the corresponding authors. The data are not publicly available due to local regulations.

## Supplementary Materials

The following supporting information can be downloaded at: https://www.mdpi.com/article/10.3390/cells11162475/s1, Figure S1: Hydroxyl radical footprinting of nucleosomes; Figure S2: Mutation H2BI36E does not inhibit FACT-facilitated transcription through the nucleosome in vitro.
