# Supplementary material for: N-Terminal Tails of Histones H2A and H2B Differentially Affect Transcription by RNA Polymerase II In Vitro
Source: Cells. 2022 Aug 10;11(16):2475. doi: 10.3390/cells11162475 (PMC9406932; doi:10.3390/cells11162475)
Supplement: Supplementary file 1 [file cells-11-02475-s001.zip › cells-1850662-supplementary.pdf]

N-terminal Tails of Histones H2A and H2B Differentially Affect Transcription by Pol II *in vitro*

Han-Wen Chang, Alexey V. Feofanov, Alexander V. Lyubitelev, Grigory A. Armeev, Elena Kotova, Fu-Kai Hsieh, Mikhail P. Kirpichnikov, Alexey K. Shaytan and Vasily M. Studitsky

Supplementary figures S1-S2

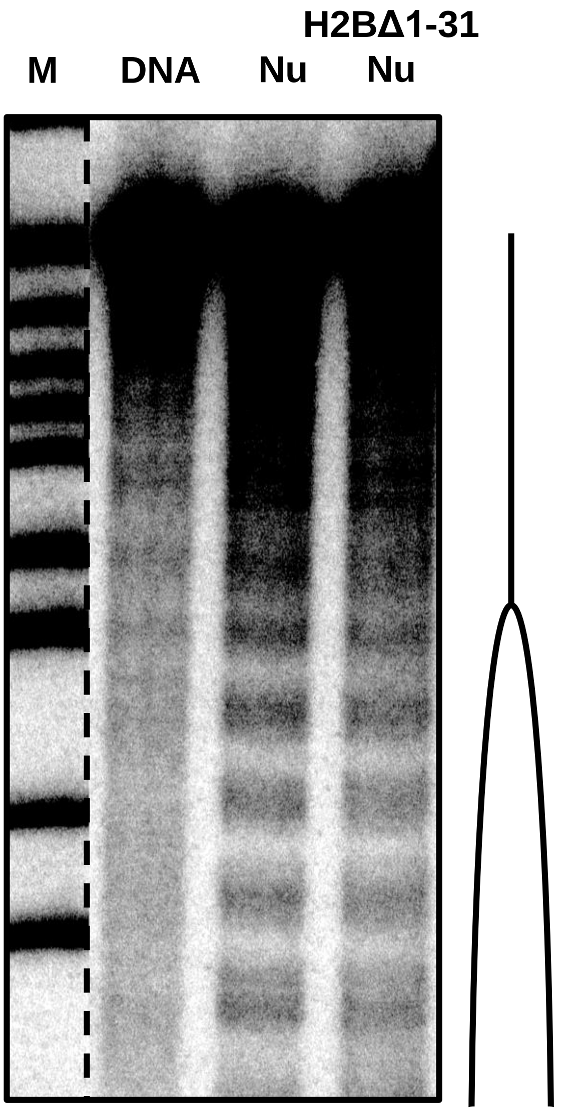

Supplementary Figure S1. Hydroxyl radical footprinting of nucleosomes. End-labeled L603 DNA and nucleosomes (Nu) containing either intact H2A/H2B or H2A/H2BΔ1-31 mutant nucleosome (H2BΔ1-31 Nu) were incubated in the presence of hydroxyl radicals and separated by native PAGE. DNA was purified from corresponding bands and analyzed by denaturing PAGE.

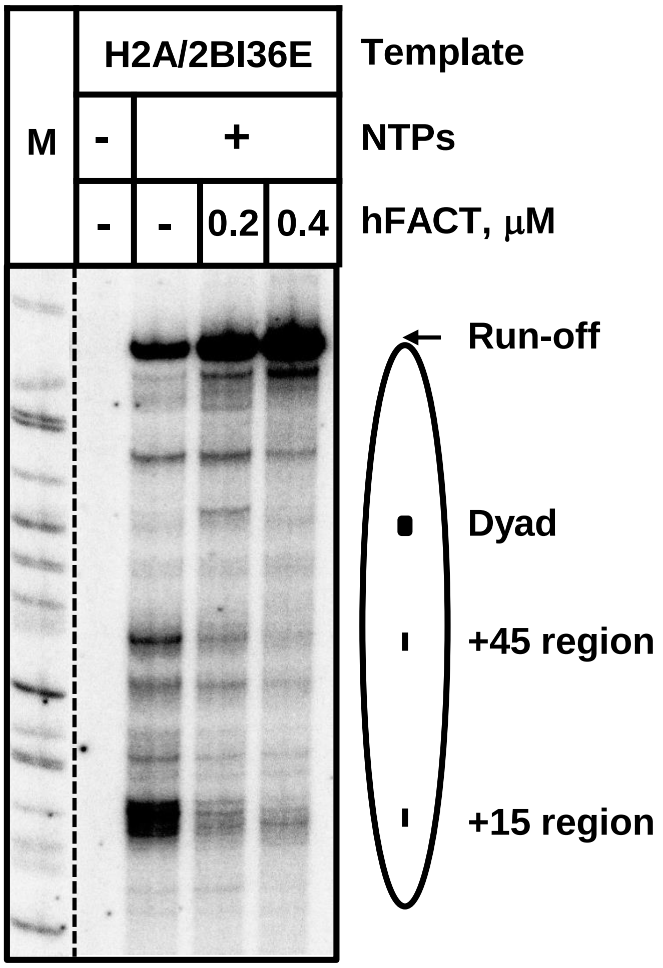

Supplementary Figure S2. Mutation H2BI36E does not inhibit FACT-facilitated transcription through the nucleosome *in vitro*. 603 nucleosome containing H2A/ H2BI36E dimers were transcribed by Pol II at 150 mM KCl in the presence or absence of human FACT protein complex. The FACT-facilitated transcription is as efficient as in the control experiment shown in Figure 5, suggesting the H2BI36E mutation strongly affecting the putative FACT-H2B interaction is not sufficient to abolish the hFACT action during Pol II transcription through the nucleosome.
